# Study on the effect and mechanism of *Lacticaseibacillus rhamnosus* AFY06 on inflammation-associated colorectal cancer induced by AOM/DSS in mice

**DOI:** 10.3389/fmicb.2024.1382781

**Published:** 2024-03-20

**Authors:** Jing Zhang, Piyun Zhang, Sijia Li, Ting Yu, Xiangyu Lai, Yongpeng He

**Affiliations:** ^1^Environmental and Quality Inspection College, Chongqing Chemical Industry Vocational College, Chongqing, China; ^2^Department of Gastroenterology and Hepatology, Chongqing Emergency Medical Center, Chongqing University Central Hospital, Chongqing, China; ^3^College of Traditional Chinese Medicine, Chongqing Medical University, Chongqing, China; ^4^Chongqing Key Laboratory of Translational Research for Cancer Metastasis and Individualized Treatment, Chongqing University Cancer Hospital, Chongqing Cancer Institute, Chongqing Cancer Hospital, Chongqing, China

**Keywords:** *Lacticaseibacillus rhamnosus*, colon cancer, azoxymethane, mRNA, mice

## Abstract

**Introduction:**

*Lacticaseibacillus rhamnosus* AFY06 (LR-AFY06) is a microorganism isolated from naturally fermented yogurt in Xinjiang, China.

**Methods:**

In this study, we investigated the effects and mechanisms of LR-AFY06 in a mouse model of inflammation-associated colon cancer. The mouse model was established by azoxymethane/dextran sulfate sodium (AOM/DSS) induction. The tumor number in intestinal tissues was counted, and the histopathological analysis was performed on colon tissues. Enzyme-linked immunosorbent assay and real-time quantitative polymerase chain reaction were performed to measure relevant protein levels in colon tissues.

**Results:**

LR-AFY06 treatment alleviated weight loss, increased organ index, reduced intestinal tumor incidence, improved histopathological damage, decreased the levels of inflammatory cytokines such as interleukin-6 (IL-6), interleukin-1 beta (IL-1β), tumor necrosis factor alpha (TNF-α), nuclear factor κB (NF-κB), and inducible nitric oxide synthase (iNOS) in the serum and colon tissue, downregulated the mRNA expression of inhibitor of NF-κB beta (IκBβ), p65, p50, p52, B-cell lymphoma-2 (Bcl-2), and B-cell lymphoma-extra large (Bcl-xL) in colon tissues, and increased the mRNA expression of Bid and caspase-8. The high concentration of LR-AFY06 exerted a better effect than the low concentration; however, the effect was slightly inferior to that of aspirin. Moreover, LR-AFY06 mitigated the intestinal inflammatory process and inhibited intestinal tumor development by regulating the NF-κB and apoptosis pathways.

**Discussion:**

The present study indicates the regulatory potential of LR-AFY06 in inflammation-associated colorectal cancer in mice, providing a valuable basis for further research.

## 1 Introduction

Colorectal cancer, one of the most common malignancies, is the third leading cause of cancer worldwide, with high incidence and mortality rates. Furthermore, it affects younger individuals more than adults, posing a major threat to human health (Sung et al., 2021). The complex and diverse pathogenesis of colorectal cancer involves environmental and dietary factors, personal habits, and genetic factors such as family history and hereditary factors (Thanikachalam and Khan, 2019). Additionally, chronic inflammation is a critical risk factor for various cancers, and the severity of chronic intestinal inflammation is positively correlated to the occurrence of intestinal tumors, which eventually may develop into inflammation-related colorectal cancer (Axelrad et al., 2016; Pang et al., 2018). Various experimental animal models of colorectal cancer have been developed to investigate the pathological mechanisms of colorectal cancer and simulate the pathological process of human colon cancer. Among them, the azoxymethane (AOM) and dextran sodium sulfate (DSS) model is a simple and highly reproducible animal model of colorectal cancer (Clark and Starr, 2016). The carcinogen AOM can induce tumor formation, whereas the sulfated polysaccharide DSS, which is similar to heparin, can damage colonic epithelial cells, resulting in intestinal inflammation (Parang et al., 2016). High precision and short cycle are the main characteristics of the AOM/DSS model, with colorectal tumors occurring and developing within as short as 10 weeks. Furthermore, the histopathology of tumor tissues of AOM/DSS closely mimics the occurrence and development of human colorectal cancer (Eichele and Kharbanda, 2017).

The pathophysiological mechanisms of colorectal cancer are complex, with a multistage development process. The nuclear factor κB (NF-κB) signaling pathway plays a predominant role in the pathophysiology of colorectal cancer, affecting tumor initiation, progression, and metastasis (Martin et al., 2021). NF-κB is a major link between inflammation and cancer; thus, it plays a crucial role in colorectal cancer occurrence and development through its mediated transcription. NF-κB signaling activation promotes the establishment of a pro-inflammatory tumor microenvironment in colorectal cancer cells and regulates cell proliferation, apoptosis, metastasis, angiogenesis, drug resistance, and inflammation-related target gene expression (Hu et al., 2022). Simultaneously, NF-κB is a major anti-apoptotic factor. It activates anti-apoptotic proteins (B-cell lymphoma [Bcl-2] and Bcl-xL), inactivates pro-apoptotic proteins (Bid, Bax, and Bak), and reduces caspase activity, thereby inhibiting the apoptosis of colorectal cancer cells (Wang et al., 2009).

Currently, fluorouracil, irinotecan, oxaliplatin, and cetuximab are some commonly used therapeutic drugs for patients with colorectal cancer; however, these drugs often exhibit certain side effects (Amelimojarad et al., 2022). Aspirin, a clinical anti-inflammatory drug, has garnered considerable attention owing to the close relationship between colorectal cancer and inflammation. Aspirin exerts a preventive effect on cancer, particularly colorectal cancer (Bosetti et al., 2020). Moreover, the U.S. Preventive Services Task Force published a draft recommendation in 2015 regarding aspirin usage in the primary prevention of colorectal cancer (Drew et al., 2016), presenting aspirin as a choice for intervention in inflammation-related colorectal cancer. However, clinical studies have shown that aspirin exhibits gastrointestinal side effects, particularly damage to the upper digestive tract, including dyspepsia and peptic ulcer bleeding, which may lead to death (Li et al., 2020). Compared to treatment with drugs exhibiting multiple side effects, dietary intervention to manage colorectal cancer occurrence and development has become a research hotspot. Among them, isolating and selecting probiotic strains from fermented dairy products to develop functional foods with more effective outcomes are of great importance in colorectal cancer management.

Probiotics can maintain the intestinal health of the host and slow down gastrointestinal cancer occurrence to some extent (Fotiadis et al., 2008). In China, Xinjiang is inhabited by numerous ethnic minorities who rely on its unique geographical, environmental, and climatic conditions, diverse livestock farming, and abundant raw milk to provide ample raw materials for pastoralists to manually process various dairy products. Specifically, in Xinjiang, nomadic ethnic groups have been living for generations, and they have retained the habit of homemade fermented milk, which exhibits distinctive regional characteristics and unique flavors (Zhao et al., 2019). These fermented dairy products contain abundant lactic acid bacteria, which are natural probiotics with tremendous research and application value. *Lacticaseibacillus rhamnosus*, a major category of lactic acid bacteria, is widely found in fermented dairy products, meat, and vegetables. Moreover, it is a probiotic found in the human gastrointestinal tract, and it exerts remarkable promoting effects on human health (Garcia-Gonzalez et al., 2021). Additionally, it is an edible lactic acid bacterium. *Lacticaseibacillus rhamnosus* affects colorectal cancer occurrence and development by regulating immunity and cell stress response, inhibiting inflammation, and regulating cell proliferation, apoptosis, necrosis, and metastasis (Javid et al., 2023). Additionally, it exerts certain protective effects on normal cells and the body. There is currently limited research on the therapeutic effect of *Lacticaseibacillus rhamnosus* on tumors. Only a few studies have shown that *Lacticaseibacillus rhamnosus* has an intervention effect on Caco-2 colon cancer cells, while derivative studies have shown that the metabolites of *Lacticaseibacillus rhamnosus* have an apoptotic induction effect on colon cancer cells (Cui et al., 2011; Shi et al., 2023).

The Xinjiang region of China is adjacent to Central Asia, with a unique geographical environment and a multi-ethnic mixed population. The microbial diversity of naturally fermented yogurt is good. Our team isolated and identified the microorganisms in Xinjiang’s naturally fermented yogurt after sampling, and selected *Lacticaseseibacillus rhamnosus* AFY06 (LR-AFY06) with good *in vitro* resistance. Therefore, LR-AFY06 was selected for subsequent experiments in this study. Therefore, in the present study, a mouse model of inflammation-related colon cancer was established using the chemical inducers AOM and DSS. The effects and specific mechanisms of LR-AFY06 intervention on colon cancer were thoroughly investigated by characterizing the disease in the mouse model, analyzing histopathology, measuring pro-inflammatory cytokine levels, and determining inflammatory and apoptotic signaling pathways. We aimed to provide scientific evidence for developing probiotics as functional foods for targeting colon cancer and to screen candidate strains, offering a scientific basis for future studies.

## 2 Materials and methods

### 2.1 Strain

The strain LR-AFY06 was isolated from naturally fermented sour milk obtained from the homes of herders in the Altay region of Xinjiang, China. LR-AFY06 was independently isolated and identified by our team. Firstly, the microorganisms were purified using plate streaking, followed by preliminary identification using Gram staining, and finally, the species of the isolated microorganisms were identified using 16S rDNA technology. Finally, it is preserved at the China General Microbiological Culture Collection Center (CGMCC, Beijing, China) under the accession number CGMCC No. 27366.

### 2.2 Animal experimental design

C57BL/6 mice (specific pathogen-free grade, 25 ± 2 g, male, 6 weeks old) were purchased from Hunan Slake Jingda Experimental Animal Co., Ltd. (Animal License No. SCXK (Xiang) 2019-0004). After a 7-day adaptation period in a controlled environment of constant temperature and humidity, the mice were randomized into five groups as follows: normal, model, aspirin, LR-AFY06 low-concentration (LR-AFY06L), and LR-AFY06 high-concentration (LR-AFY06H) groups. During the experiment, the mice had *ad libitum* access to food and water. On the first day of modeling, mice in the model, aspirin, and LR-AFY06 groups were intraperitoneally injected with AOM (Sigma, Shanghai, China) at a concentration of 10 mg/kg, followed by the administration of the 2.5% DSS solution (Solarbio Life Sciences, Beijing, China) for 2 weeks, starting from the second, fifth, and eighth week, respectively. Mice in the normal group received no special treatment and were orally administered sterile physiological saline daily. Mice in the model group were orally administered sterile physiological saline daily. According to the clinical dosage of aspirin, which ranges from 0.3 to 0.5 g, a median value of 0.4 g was selected and converted into a mouse dosage. Mice in the aspirin group were orally administered the aspirin solution at a concentration of 67 mg/kg. According to the recommendations of the World Health Organization, adults need to consume at least 1 × 10^9^ CFU/kg per day, totaling 1.7 × 10^7^ CFU/kg per day, which is equivalent to a dose of 1.53 × 10^8^ CFU/kg per day for mice. Therefore, this study selected two doses, 1 × 10^8^ and 1 × 10^9^ CFU/kg per day, for the experiment. Mice in the LR-AFY06L and LR-AFY06H groups were orally administered the LR-AFY06 bacterial suspension at dosages of 1 × 10^8^ colony-forming units (CFU)/kg and 1 × 10^9^ CFU/kg, respectively, for 10 weeks. During the experiment, the mice were observed for any abnormal signs, and their body weights were measured and recorded weekly. After modeling, the mice were euthanized by eyeball blood collection and cervical dislocation. The colon tissues of the mice were dissected for further analysis.

### 2.3 Organ index and colon index determination

The colon tissues of the mice were weighed, and the organ index was calculated us-ing the following formula: Visceral index = visceral weight (g)/mouse body weight (g) × 100. The length of the mouse colon was measured, and the occurrence of intestinal tumors was observed. The colon index was calculated using the following formula: colon coefficient = colon weight (mg)/colon length (cm).

### 2.4 Pathological observation of colon tissues

Colon tissues, approximately 0.5 cm in length, were immersed in a 4% paraformaldehyde fixing solution for 48 h. After dehydration with ethanol, the tissues were embedded in paraffin. Approximately 2–3-μm thick sections were obtained using a microtome. The sections were then stained with hematoxylin and eosin (H&E, Solarbio Life Sciences) and mounted on glass slides to prepare the histopathological slides of mouse colon tissues. Morphological changes were observed using a light microscope (BX53, Olympus, Tokyo, Japan).

### 2.5 Determination of inflammatory biomarker levels in mouse serum and colon tissues

Mouse whole blood samples were collected and centrifuged at 4°C and 4000 rpm for 10 min to obtain serum. Following the instructions provided along with an enzyme-linked immunosorbent assay (ELISA) kit (Shanghai Enzyme-linked Biotechnology Co., Ltd., Shanghai, China), the levels of IL-6, IL-1β, TNF-α, NF-κB, and iNOS were measured in the mouse serum and colon tissues.

### 2.6 Determination of the mRNA expressions of inflammation and apoptosis-related genes in mouse colon tissues

Approximately 50 mg of mouse colon tissues were weighed and total RNA was extracted using the TRIzol reagent (Solarbio Life Sciences). The extracted RNA was then reverse-transcribed into complementary DNA (cDNA) following the instructions provided along with a cDNA synthesis kit. The concentration and purity of RNA and cDNA were measured using a microspectrophotometer. Real-time polymerase chain reaction (PCR) amplification was performed using the StepOnePlus Real-Time PCR system (Thermo Fisher Scientific, Waltham, MA, USA) with the following conditions: 40 cycles of amplification at 95°C for 15 s, 60°C for 30 s, and 95°C for 15 s, followed by a 60°C extension for 60 s and a final denaturation step at 95°C for 15 s. The relative expression intensity of each gene was calculated using the 2^–ΔΔCt^ method with Eef2 as a reference gene (Thermo Fisher Scientific). Primer sequences used in this experiment are listed in Table 1.

**TABLE 1:** Primer sequences in this experiment.

Gene	Primer sequence (5′-3′)
Eef2	F: TGTCAGTCATCGCCCATGTG R: CATCCTTGCGAGTGTCAGTGA
IκBβ	F: GACATCGCATCGGCTCTTAGA R: AACGGTCACGGTGTACTTCTG
p65	F: ACTGCCGGGATGGCTACTAT R: TCTGGATTCGCTGGCTAATGG
p50	F: AGAGGGGATTTCGATTCCGC R: CCTGTGGGTAGGATTTCTTGTTC
p52	F: TGGCATCCCCGAATATGATGA R: TGACAGTAGGATAGGTCTTCCG
Bid	F: CCAGTCACGCACCATCTTTG R: GTCCATCTCGTTTCTAACCAAGT
Bcl-2	F: GAGAGCGTCAACAGGGAGATG R: CCAGCCTCCGTTATCCTGGA
Bcl-x_L_	F: AGCGTAGACAAGGAGATGCAG R: CCAAGGCTCTAGGTGGTCATTC
Caspase-8	F: CAACTTCCTAGACTGCAACCG R: TCCAACTCGCTCACTTCTTCT

### 2.7 Data analysis

All data are presented as the mean ± standard deviation. Statistical analysis for significance was performed by one-way analysis of variance using IBM SPSS 27.0 (IBM, Armonk, NY, USA). Excel 2019 (Microsoft, Redmond, WA, USA) was used for data visualization. Different letters (a-d) in the figures indicate statistically significant differences determined by Duncan’s multiple range test (*P* < 0.05).

## 3 Results

### 3.1 The effect of LR-AFY06 on the body weight of mice

**FIGURE 1:**
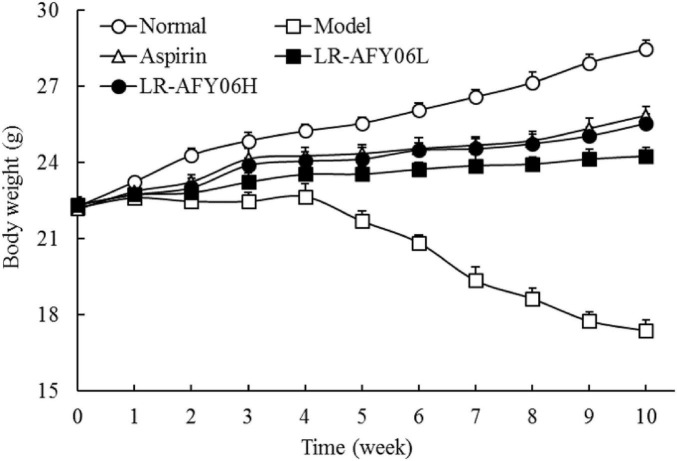
The effect of LR-AFY06 on the body weight of mice.

During modeling, mice in the normal group exhibited healthy physical appearance and normal eating and drinking habits, with no diarrhea or bloody stools. However, mice in the remaining four groups showed varying degrees of symptoms, including loose stools, bloody stools, and rectal prolapse. Additionally, these mice showed decreased activity levels, sparse and dull fur, and weight loss. The body weight of mice in the normal group increased over time, whereas those of mice in the remaining four groups decreased after each administration of the AOM/DSS solution (Figure 1). Differences in the body weights of the mice among the groups were significant (*P* < 0.05). However, after aspirin and LR-AFY06 intervention, the pathological weight loss trend in the mice was remarkably alleviated.

### 3.2 The effect of LR-AFY06 on colon length, visceral index, colon coefficient, and intestinal tumor in mice

**FIGURE 2:**
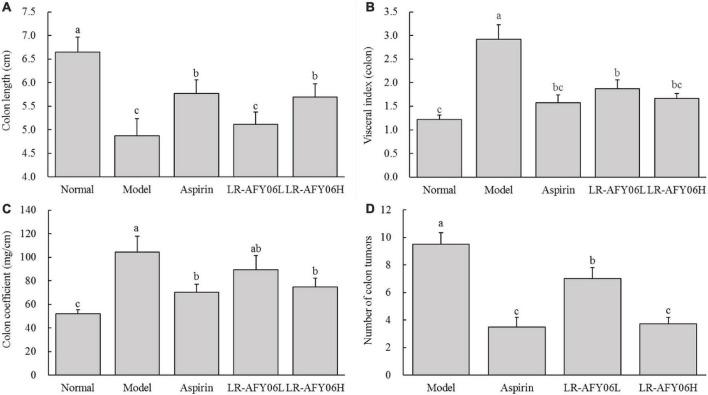
The effect of LR-AFY06 on colon tissues in mice. **(A)** Colon length; **(B)** Visceral index (colon); **(C)** Colon coefficient; **(D)** Number of colon tumors. ^a–c^Different lowercase letters indicate significant differences (*P* < 0.05).

The colon lengths of mice in the normal, model, aspirin, LR-AFY06L, and LR-AFY06H groups were 6.65 ± 0.31 cm, 4.87 ± 0.37 cm, 5.77 ± 0.29 cm, 2.12 ± 0.25 cm, and 5.69 ± 0.28 cm, respectively (Figure 2A). The colon length of mice in the model group decreased significantly compared with that of mice in the normal group (*P* < 0.05). LR-AFY06 intervention significantly alleviated colon shortening in mice (*P* < 0.05), with effects similar to those observed in the aspirin group. The visceral (colon) index and colon coefficient increased significantly in the normal, aspirin, LR-AFY06H, and LR-AFY06L groups, all of which were significantly lower than those in the model group (*P* < 0.05) (Figures 2B, C). No intestinal tumors were observed in mice in the normal group, whereas several tumors of varying sizes were found in the colon segments of mice in the model, aspirin, and LR-AFY06 groups. Mice in the model group showed the highest number of tumors (9.5 ± 0.8), whereas those in the aspirin, LR-AFY06L, and LR-AFY06H groups showed significantly reduced numbers of tumors (3.5 ± 0.7, 7.0 ± 0.8, and 3.7 ± 0.5 tumors, respectively) (Figure 2D).

### 3.3 The effect of LR-AFY06 on mouse colon tissue pathology

**FIGURE 3:**
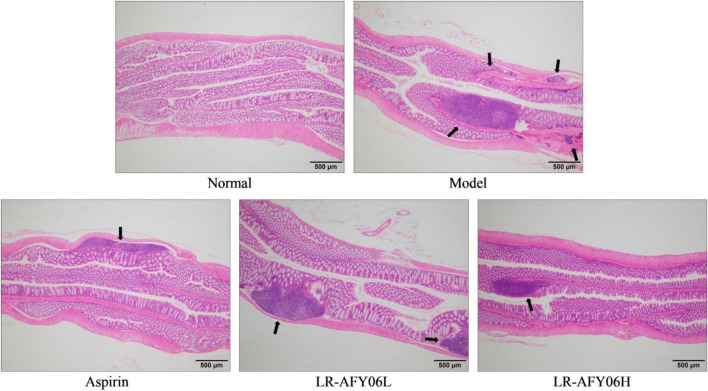
The effect of LR-AFY06 on the histopathology of mouse colon tissues (40×).

The H&E staining (Figure 3) showed that the colon tissue of mice in the normal group contained intact mucosal epithelial cells, normal crypts, well-arranged glands, and no ulcers. Mice in the model group showed an extensive infiltration of inflammatory cells in the intestines, along with multiple areas of necrotic lesions and crypt abscesses. Mice in the aspirin group showed a lower degree of inflammatory cell infiltration and less disruption of crypt structures. Although mild inflammation infiltration was observed in mice in the LR-AFY06 groups, crypt structures were relatively intact. LR-AFY06H exhibited a greater extent of improvement than did LR-AFY06L on the pathological damage of the colon tissue caused by AOM/DSS.

### 3.4 The effect of LR-AFY06 on the levels of inflammatory cytokines in mouse serum and colon tissue

**FIGURE 4:**
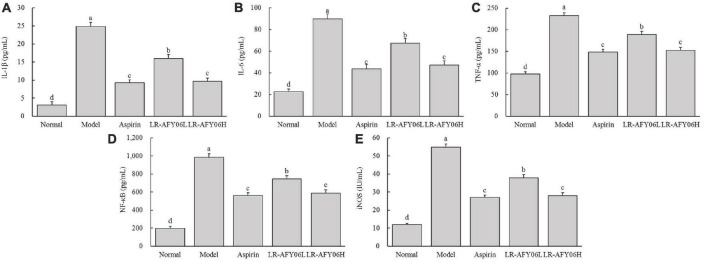
The effect of LR-AFY06 on the serum levels of inflammatory cytokines in mice. **(A)** IL-1β; **(B)** IL-6; **(C)** TNF-α; **(D)** NF-κB; **(E)** iNOS. ^a–d^Different lowercase letters indicate significant differences (*P* < 0.05).

**FIGURE 5:**
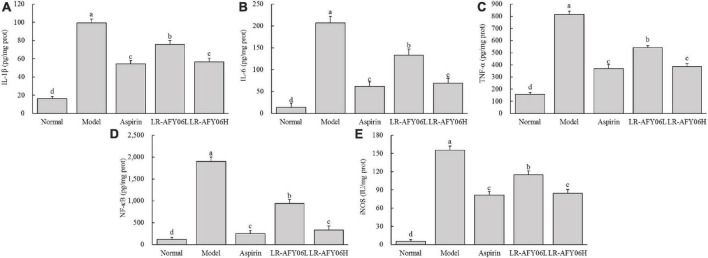
The effect of LR-AFY06 on the colon tissue levels of inflammatory cytokines in mice. **(A)** IL-1β; **(B)** IL-6; **(C)** TNF-α; **(D)** NF-κB; **(E)** iNOS. ^a–d^Different lowercase letters indicate significant differences (*P* < 0.05).

After AOM/DSS induction, the levels of IL-1β, IL-6, and TNF-α in the serum and colon of mice in the model group increased significantly (Figures 4A–C, 5A–C). Aspirin and LR-AFY06 effectively reduced their gene expression. The serum levels of IL-1β, IL-6, and TNF-α in the LR-AFY06H group were significantly lower than those in the LR-AFY06L group (*P* < 0.05), which indicated that LR-AFY06H more significantly reduced the levels of the pro-inflammatory cytokines in the mouse serum than did LR-AFY06L. The NF-κB level was highest in the serum and colon tissue of mice in the model group (Figures 4D, 5D). NF-κB levels in the serum of mice in the normal, aspirin, and LR-AFY06 groups were significantly lower than that in mice in the model group (*P* < 0.05). The iNOS gene was highly expressed in the serum and colon tissue of mice in the model group, and its levels in the serum of mice in the normal, aspirin, and LR-AFY06 groups were significantly lower than that in mice in the model group (*P* < 0.05) (Figures 4E, 5E).

### 3.5 The effect of LR-AFY06 on the mRNA expressions of inflammation pathway-related factors in mouse colon tissue

**FIGURE 6:**
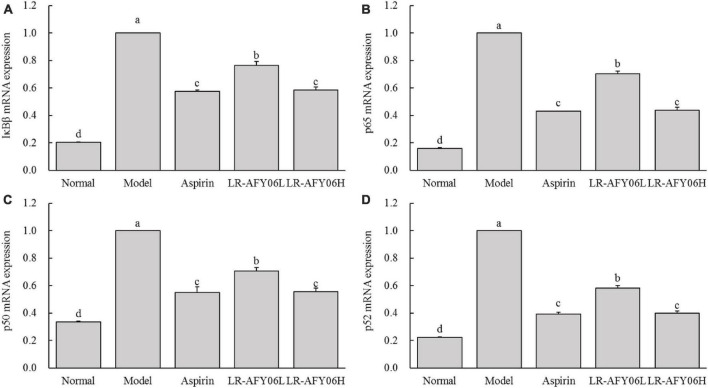
The effect of LR-AFY06 on the relative mRNA expressions of inflammatory factors in mouse colon tissues. **(A)** Relative expression of IκBβ; **(B)** Relative expression of p65; **(C)** Relative expression of p50; **(D)** Relative expression of p52. ^a–d^Different lowercase letters indicate significant differences (*P* < 0.05).

Real-time fluorescence quantitative PCR was performed to analyze the mRNA expressions of IκBβ, p65, p50, and p52 in the mouse colon tissues, and the results showed (Figure 6) that their mRNA expressions in the colon tissues of mice in the model group were highest, whereas that in the aspirin and LR-AFY06 groups decreased significantly (*P* < 0.05). Moreover, the effect of oral LR-AFY06H administration was comparable to that of aspirin administration.

### 3.6 The effect of LR-AFY06 on the mRNA expressions of apoptosis pathway-related factors in mouse colon tissue

**FIGURE 7:**
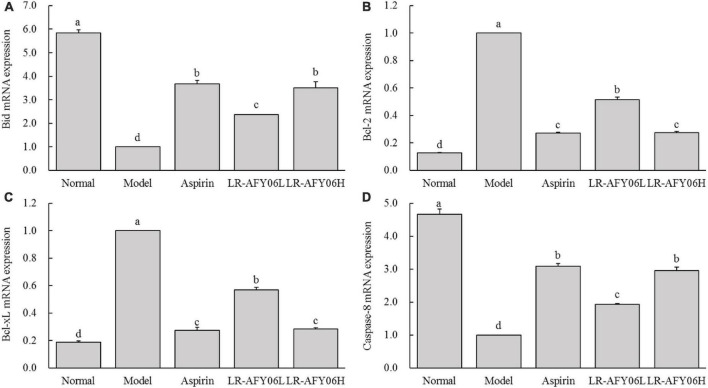
The effect of LR-AFY06 on the relative mRNA expressions of apoptosis-related factors in mouse colon tissues. **(A)** Relative expression of Bid; **(B)** Relative expression of Bcl-2; **(C)** Relative expression of Bcl-xL; **(D)** Relative expression of caspase-8. ^a–d^Different lowercase letters indicate significant differences (*P* < 0.05).

Compared with mice in the normal group, those in the model group showed downregulated Bid (pro-apoptotic factor) mRNA expression; however, its relative expressions were significantly upregulated in mice in the aspirin and LR-AFY06 groups (Figure 7A) (*P* < 0.05). Furthermore, Bcl-2 and Bcl-xL (anti-apoptotic factors) mRNA expression was upregulated in mice in the model group (Figures 7B, C) and was significantly downregulated in the aspirin and LR-AFY06 groups (*P* < 0.05). Compared with mice in the normal group, AOM/DSS-induced mice in the model group showed significantly downregulated caspase-8 (pro-apoptotic factor) mRNA relative expression in colon tissues (*P* < 0.05) (Figure 7D). However, aspirin and LR-AFY06 treatment significantly improved caspase-8 mRNA downregulation caused by carcinoma in the mice. Compared with mice in the model group, those in the LR-AFY06L and LR-AFY06H groups showed significant differences in caspase-8 mRNA expression (*P* < 0.05). Thus, LR-AFY06 significantly upregulated Bid and caspase-8 and downregulated Bcl-2 and Bcl-xL mRNA expression.

## 4 Discussion

The chemically-induced AOM/DSS mouse model of colon cancer reliably reproduces the stages of intestinal tumor occurrence and development, closely mimicking the pathological conditions of human colon cancer. This chemical induction can lead to weight loss in cancer-afflicted mice (De Robertis et al., 2011). Colon length is one of the indicators used to characterize the severity of DSS-induced colonic inflammation (Min et al., 2015). The organ coefficient is a fundamental parameter and an essential basis in biomedical research. Inflammation and edema can increase body weight and organ coefficient (Michael et al., 2007; Xu et al., 2023). AOM/DSS used in this study induced tumors and promoted tumor activity (Erben et al., 2014). After successful modeling, LR-AFY06 could effectively alleviate colon shortening caused by weight loss, intestinal inflammation and edema, reduced colonic coefficient and visceral index in mice, and reduced intestinal tumorigenesis, which had interventional potential in colon cancer.

Cytokines, the main signaling molecules released by inflammatory cells, play roles in multiple functions. The major pro-inflammatory cytokines include IL-1β, IL-6, and TNF-α, the continuous intensification of inflammation is an important cause of cancer (Mey et al., 2007; Oshima and Oshima, 2012). Herein, the anti-tumor effect of LR-AFY06 was assessed in relation to the mRNA expression of these pro-inflammatory cytokines. The mRNA levels of IL-1β, IL-6, TNF-α, NF-κB, and iNOS in the mouse serum and colon tissues were determined by performing ELISA. IL-6 activates the NF-κB signaling pathway and promotes the gene expression of cell adhesion molecules (Elahi et al., 2023). Moreover, this representative member of the interleukin family is involved in inflammation, immune regulation, and cancer development (Lopetuso et al., 2013). IL-1β stimulates inflammatory cells to enter the intestine via autocrine and paracrine mechanisms, thereby causing tissue damage and inflammation (He et al., 2023). Remarkably decreased IL-1β levels in a study indicated that probiotics reduce inflammatory factor levels (Wang et al., 2012). Additionally, TNF-α, a key inflammatory cytokine that regulates classical NF-κB inflammatory pathway activation, acts as both an activator and a product of NF-κB activation (Lopetuso et al., 2013; Cotino-Nájera et al., 2023). Hence, TNF-α and NF-κB are often studied together. NF-κB, a key mediator of the inflammatory response, is one of the most common regulatory factors in cancer. Changes in NF-κB levels reflect the disease status in patients with colon cancer (Alipourgivi et al., 2023). Additionally, NF-κB activation can increase iNOS levels in the tissue. Moreover, TNF-α activates iNOS as part of the inflammatory response (Mey et al., 2007). iNOS is related to tumor occurrence and development and serves as a novel target for cancer prevention and treatment (Zhao et al., 2021). The present results revealed that AOM/DSS application induced the high expression of the pro-inflammatory cytokines and iNOS in the mice, which eventually activated the NF-κB inflammatory pathway and resulted in sustained inflammation in the body. LR-AFY06 intervention effectively decreased IL-1β, IL-6, and TNF-α levels in the mice, downregulated NF-κB and iNOS mRNA expression, and alleviated the pathological state of inflammation in both the serum and colon tissues.

The NF-κB signaling pathway activated in mice after AOM/DSS induction increases the level of inflammation. Thus, such mice can be protected from inflammatory stimuli and colon cancer occurrence and development can be intervened by inhibiting the NF-κB signaling pathway. IκBβ, a member of the IκB protein family, plays a dual regulatory role in the NF-κB pathway. It forms an IκBβ:p65:cRel complex to promote the transcription of target genes, thereby activating the NF-κB pathway (Kamata et al., 2010). Conversely, free NF-κB plays a crucial regulatory role in the cytoplasm and nucleus. The phosphorylated subunit p65 triggers the transcription of certain genes, such as TNF-α, IL-1β, and IL-6. Subsequently, these cytokines promote the activation of the Toll-like receptor 4-mediated pathway, thereby enhancing and amplifying the inflammatory response (Takeuchi and Akira, 2010). Additionally, p65 and p50, two important subunits of NF-κB, are important biomarkers for colon cancer diagnosis and prognosis. Moreover, the p52/RelB dimer promotes the transcription of inflammation-related genes via nuclear translocation (Martin et al., 2021).

The NF-κB signaling pathway promotes the establishment of an inflammatory tumor microenvironment in colon cancer, which can serve as a source of tumor markers for several processes, such as cell survival, proliferation, metastasis, and angiogenesis. In the tumor microenvironment, NF-κB upregulates the gene expression of anti-apoptotic proteins, such as Bcl-2 and Bcl-xL (Martin et al., 2021). Bid, a pro-apoptotic member of the Bcl-2 family, induces mitochondrial apoptosis in colon cancer (Huang et al., 2017). Furthermore, Bcl-2 and Bcl-xL expression inhibits the normal apoptotic program of tumor cells and promotes their survival and development. Therefore, targeting apoptosis-related factors is an effective approach to treat colon cancer. Additionally, caspase-8, an essential part of the regulation and initiation of death receptor-mediated programmed cell death, is closely associated with intrinsic and extrinsic apoptotic pathways. The dysfunction of caspase-8 may contribute to malignant tumor development in mice and humans (Tummers and Green, 2017). The present results showed that LR-AFY06 effectively regulated the expression of inflammation and apoptosis-related genes in the colon cancer tissues of the mice, thus exerting preventive and inhibitory effects.

Colon cancer has a great impact on the human intestine, affecting the absorption of nutrients, causing difficulty in defecation, but also intestinal dysfunction, intestinal flora disorder, comprehensive impact on the intestinal flora involved in the human immune regulation, inflammation and oxidative stress response (Zhang et al., 2024). Studies have shown that *lactococcus lactis*, *Lactobacillus acidophilus* and *Bifidobacterium* can interfere with the development of colon cancer by regulating immune function and inflammatory response (Li and Li, 2003; Agah et al., 2019). A study has shown that some *Lactobacillus rhamnosus* can regulate intestinal function, have a regulatory effect on intestinal barrier function, can intervene in the development of diseases by regulating immune function, and has probiotic function (Lebeer et al., 2010). But, there are few studies on the effect of *Lacticaseibacillus rhamnosus* on colon cancer. This study is the first to study the intervention effect of *Lacticaseibacillus rhamnosus* AFY06. The results showed that LR-AFY06 significantly improved symptoms such as weight loss, increased organ index, shortened colon length, and increased intestinal index, thereby effectively reducing the incidence of intestinal tumors in the cancer-bearing mice and improving pathological damage that occurred in colon tissues (*P* < 0.05). Furthermore, LR-AFY06 intervention significantly decreased the levels of the inflammatory cytokines IL-1β, IL-6, TNF-α, NF-κB, and iNOS in the serum and colon tissue of the mice (*P* < 0.05). Additionally, at the gene level, LR-AFY06 significantly downregulated the mRNA expression of the pro-inflammatory factors IκBβ, p65, p50, and p52 and the anti-apoptotic factors Bcl-2 and Bcl-xL in the colon tissues, whereas it upregulated the mRNA expression of the pro-apoptotic factors Bid and caspase-8 (*P* < 0.05). The effect of *Lacticaseibacillus rhamnosus* on the regulation of colon cancer by regulating the mechanism of inflammation has been preliminically studied, which can further promote the probiotic effect of *Lacticaseibacillus rhamnosus*. However, this study is a preliminary animal experimental study, and the mechanism of intestinal regulation of *Lacticaseibacillus rhamnosus* in the development of colon cancer needs further study. The mechanism of action in humans also needs to be verified in clinical trials.

## 5 Conclusion

We performed a comprehensive study on the effects and mechanisms of LR-AFY06, isolated from traditional naturally fermented acidophilus milk in Xinjiang, on inflammation-related colon cancer using an AOM/DSS-induced murine model. The evaluation was based on five aspects, namely the body weight and organ index of the mice, colon index and number of intestinal tumors, pathological analysis of colon tissues, analysis of inflammation-related cytokines in serum and colon tissues, and analysis of the expression of genes related to NF-κB and cell apoptosis signaling pathways in colon tissues. We are the first to demonstrate the significant effects of LR-AFY06 intervention on the occurrence and development of inflammation-related colon cancer in mice and reveal its specific mechanism of slowing down colon cancer development by improving intestinal inflammation and promoting intestinal tumor cell apoptosis. It provides scientific evidence for the functional health-related effects of LR-AFY06 on the intestine and offers a scientific basis for the prevention and adjuvant treatment of colon cancer from a dietary perspective. Further clinical research is needed to validate the present findings.

## Data availability statement

The original contributions presented in this study are included in this article/Supplementary material, further inquiries can be directed to the corresponding authors.

## Ethics statement

The animal study was approved by the Animal License No. SCXK (Xiang) 2019-0004. The study was conducted in accordance with the local legislation and institutional requirements.

## Author contributions

SL: Writing – original draft. TY: Writing – original draft. JZ: Writing – original draft. PZ: Writing – original draft. XL: Writing – review and editing. YH: Writing – review and editing.
