# Supplementary figures and images for: Study on the effect and mechanism of Lacticaseibacillus rhamnosus AFY06 on inflammation-associated colorectal cancer induced by AOM/DSS in mice
Source: Front Microbiol. 2024 Mar 20;15:1382781. doi: 10.3389/fmicb.2024.1382781 (PMC10987852; doi:10.3389/fmicb.2024.1382781)

## Slide 1
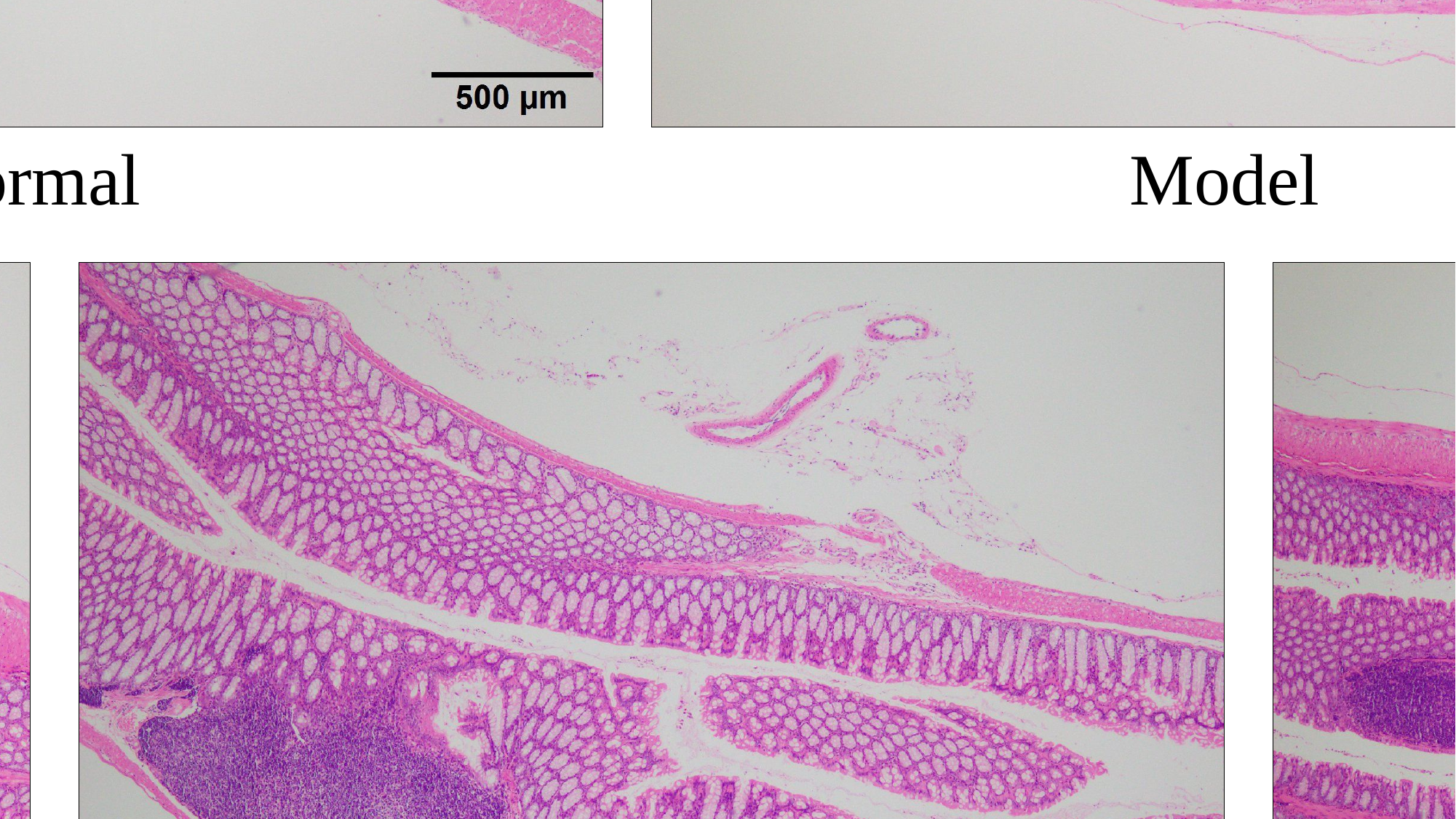

Normal
Model
Aspirin
LR-AFY06L
LR-AFY06H

Supplement: Supplementary file 1 [file Data_Sheet_1.ZIP › DATA/Fig 3.pptx]
